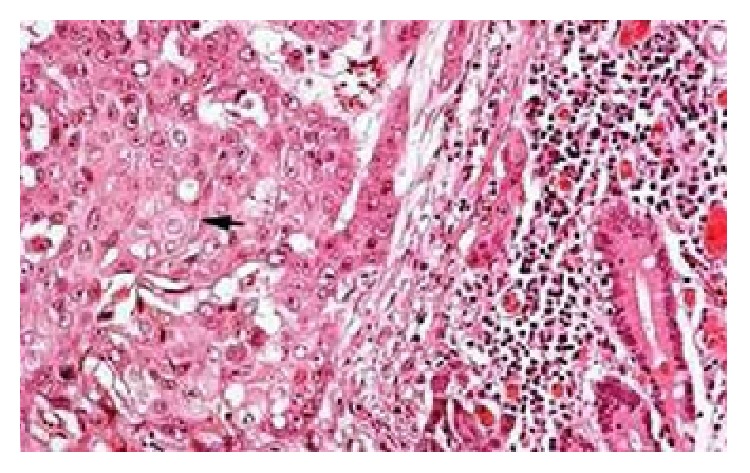# Overlap of Acute Cholecystitis with Gallstones and Squamous Cell Carcinoma of the Gallbladder in an Elderly Patient

**DOI:** 10.1155/2015/767196

**Published:** 2015-08-19

**Authors:** İhsan Yıldız, Yavuz Savas Koca, İbrahim Barut

**Affiliations:** Department of Genaral Surgery, School of Medicine, Suleyman Demirel University, Isparta, Turkey

## Abstract

*Introduction*. The incidence of gallbladder cancer presenting with acute cholecystitis is 2.3%, squamous cell carcinoma is rarely seen, and overlap of acute cholecystitis and squamous cell carcinoma is a very rare condition in the literature. *Presentation of Case*. A 75-year-old woman was admitted to emergency service with a pain in the right upper quadrant, nausea, and vomiting. The patient was considered as having acute cholecystitis. During the exploration because Hartman's pouch was not dissected, it was adhered to the cystic duct and had fibrotic adhesion. It could not be understood whether this adhesion was a tumor or a fibrotic tumor and thus we performed cholecystectomy with a 1 cm resection of the choledochus. Pathological examination revealed the presence of (R0), T1 N0 M0 squamous cell carcinoma with clean resection borders and there was no in tumor five dissected lymph nodes. The patient has been followed up for about two years and no clinical problem has been observed throughout the follow-up. *Discussion*. Acute cholecystitis with gallstones may overlap with gallbladder cancer and generally presents nonspecific symptoms. No additional imaging techniques were performed since no clinical sign except for the wall thickening was detected and no suspected malignancy such as mass was detected on USG. Squamous cell carcinoma of the gallbladder shows poor diagnosis, but since its overlap with cholecystitis presents early symptoms and thus leads to early diagnosis and effective treatment, the localization of the carcinoma is of prime importance. *Conclusion*. Gallbladder cancer should be kept in mind in elderly patients with acute cholecystitis.

## 1. Introduction

Acute cholecystitis with gallstones is the most common surgical problem of the gallbladder. The incidence of gallbladder cancer presenting with acute cholecystitis is 2.3% [1, 2]. Among the gallbladder cancers, squamous cell carcinoma is rarely seen [2]. The overlap of acute cholecystitis and squamous cell carcinoma is a very rare condition in the literature [3–6].

Gallbladder cancers, comprising 90% adenocarcinoma and 0.5–3% squamous cell carcinoma (SCC), account for 3-4% of all malignant tumors. SCC is more common at advanced ages and the female to male ratio is 1/2 [7–9]. SCC presents no specific symptom except for the clinical presentation of acute cholecystitis. Nevertheless, there are a number of studies reporting the presentation of a palpable mass in the right upper quadrant at advanced ages [8, 9].

Early diagnosis of SCC accounts for 10–50% of the cases. Ultrasonography (USG) is the method of choice in the diagnosis but advanced techniques such as tomography are also used, particularly for the suspected cases. In the diagnosis of gallbladder cancer, the sensitivity of USG is 34%, whereas it is ~40 for tomography [8].

Surgical treatment of SCC is followed by radiotherapy and chemotherapy. Early stages of SCC are treated by cholecystectomy and the late stages are treated by extensive hepatectomy including resection of segment 4 or 5 [8, 9].

Survival rate following surgery is 20% in the early-stage patients and 10% in the late-stage patients [9].

In this study, we present a 75-year-old female patient who presented to our emergency service with a pain in the right upper quadrant and underwent cholecystectomy due to acute cholecystitis and then presented with squamous cell carcinoma of the gallbladder in the pathological examination.

## 2. Case

A 75-year-old woman presented to our emergency service with a pain in the right upper quadrant, nausea, and vomiting. The patient had no clinical features and had no specific sign or symptom except for a temperature of 37.5°C and a positive Murphy's sign in the right upper quadrant.

Biochemical parameters were as follows: BK: 12,000 (4,000–8,000), AST: 45 (0–35 U/L), ALT: 56 (0–35 U/L), and AMLZ: 120 (24–151 U/L). The other parameters were normal.

Abdominal USG revealed swollen gallbladder with a wall thickness of 2 cm and echogenicity suggesting a 3 cm stone in Hartman's pouch. Depending on these signs, the patient was considered as having acute cholecystitis.

## 3. Intraoperative Findings

The surgery started with laparoscopy. During the exploration, the gallbladder was swollen, the wall was thickened and inflamed, pericholecystic minimal fluid was detected, and the other intra-abdominal organs were normal. The gallbladder was suspended from the fundus. The Calot's triangle was dissected but it was difficult to dissect the Hartman's pouch since it was adhered to the cystic duct and to the 1 cm segment of the choledochus. We had no available conditions for frozen biopsy during the surgery.

Afterwards, the procedure was converted to open surgery, and cholecystectomy was performed by the resection of 1 cm segment of the choledochus (because Hartman's pouch was not dissected since it was adhered to the cystic duct and had fibrotic adhesion). The choledochus was primarily anastomosed over the T-tube. The patient was uneventfully discharged on postoperative day 6. On postoperative day 14, a T-tube cholangiography was performed and no clinical problem was observed. The T-tube was removed on postoperative day 21.

**Figure 1:**
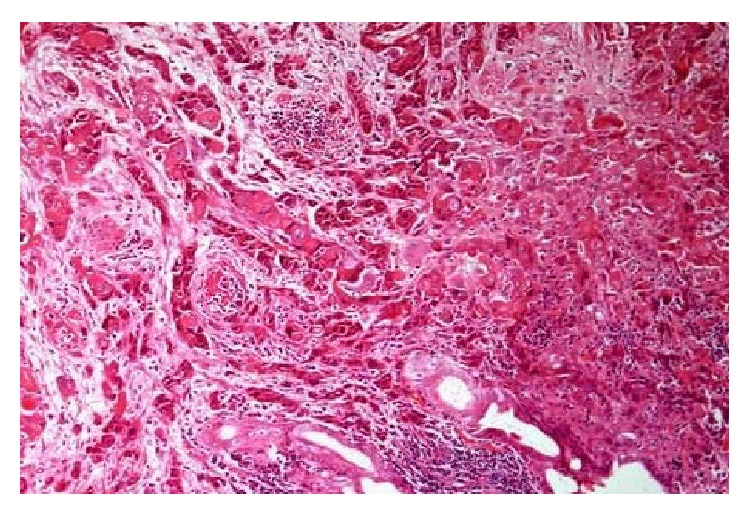


Pathological examination revealed the presence of (R0), T1 N0 M0 squamous cell carcinoma with clean resection borders and there was no tumor in five dissected lymph nodes (Figures 1 and 2).

No additional treatment was performed since the patient refused to undergo any treatment after surgery. The patient has been followed up for about two years and no clinical problem has been observed throughout the follow-up.

## 4. Discussion

Acute cholecystitis is the most common surgical problem of the gallbladder. Acute cholecystitis mainly results from gallstones and also may arise from a tumor which blocks the bile flow [2, 3].

Acute cholecystitis with gallstones may overlap with gallbladder cancer. The incidence of this overlap is reported to be 2.3% [9]. The female to male ratio is 1/2 [7–9]. Gallbladder cancers comprise 90% adenocarcinoma and 0.1–7% SCC [7, 9].

Acute cholecystitis presents no specific symptom suggestive of a tumor. Nevertheless, a palpable mass may be presented in the right upper quadrant at advanced ages [7–9].

Acute cholecystitis generally presents nonspecific symptoms including a pain in the right upper quadrant, nausea, and vomiting and the diagnosis is often achieved by USG. Most common USG finding is wall thickening, which is detected in 70–100% of the cases. Further diagnosis of suspected cases is achieved by Computed Tomography (CT), Magnetic Resonance Imaging (MRI), Percutaneous Transhepatic Cholangiography (PTC), and Magnetic Resonance Cholangiopancreatography (MRCP). Similarly, our case presented with a pain in the right upper quadrant, nausea, and vomiting. However, no additional imaging techniques were performed since no clinical sign except for the wall thickening was detected and no suspected malignancy such as mass was detected on USG.

The treatment of acute cholecystitis with gallstones is performed via two approaches: early stage and late stage (with antibiotic therapy). In our patient, we preferred the early-stage approach considering that the late-stage approach would worsen the general condition of the patient. Moreover, we preferred this approach since we had no gastroenterology department in our hospital that would perform the follow-up of the patient.

Literature shows that if a tumor is detected in the gallbladder during the surgery, either the treatment should be continued and the definitive diagnosis should be achieved via frozen biopsy or the surgery should be terminated and the staging of the tumor should be performed [8]. However, we preferred to continue the surgery since we had no available conditions for frozen biopsy. Most common USG finding is wall thickening, which is detected in 70–100% of the cases. Further diagnosis of suspected cases is achieved by Computed Tomography (CT), Magnetic Resonance Imaging (MRI), Percutaneous Transhepatic Cholangiography (PTC), and Magnetic Resonance Cholangiopancreatography (MRCP). Nevertheless, no surgical deficit occurred in our patient since no different surgical procedure is suggested when a tumor is detected on frozen biopsy. Surgery is the method of choice in the treatment. The first and second stages of SCC are treated by cholecystectomy, whereas the late stages are treated by extensive cholecystectomy with wedge resection and hepatectomy including resection of segment 4 or 5 [8].

In our patient, the Hartman's pouch was not dissected since it was adhered to the cystic duct and to the 1 cm segment of the choledochus. Nevertheless we could not understand whether this adhesion was a tumor or a fibrotic tumor and thus we performed cholecystectomy with a 1 cm resection of the choledochus and choledochocholedochostomy over the T-tube.

Squamous cell carcinoma of the gallbladder has a poor prognosis since it is a rare condition and leads to delayed diagnosis [2, 3, 6, 8]. The involvement of serosal and lymphatic glands is a major factor for the poor prognosis. SCC is mostly diagnosed in the wall (25%), through the involvement of the lymphatic gland (35%), and through distant metastasis (40%) [8, 9]. In the cases with lymphatic gland involvement, the prognosis remains poor despite extensive surgery [9]. The 5-year survival rate is 20% after surgery, whereas it is 0% in the nonoperated patients and 10% in the late-stage patients undergoing surgery. In stages 2 and 3, the 5-year survival rate is 0% after cholecystectomy and 29% after extensive cholecystectomy [3, 9].

Radiotherapy and chemotherapy are suggested after the surgical treatment of SCC. However, our patient refused to undergo any of these treatments. Nevertheless, the patient has been followed up for about two years and no clinical problem has been observed throughout the follow-up.

Gallbladder cancer, as in most cancers, is more common in advanced ages. Gallbladder cancer is more frequent in women than in men and it generally presents nonspecific symptoms such as acute cholecystitis [1–3, 7, 8]. Similarly, our patient was 75 years old and she presented no clinical sign suggestive of gallbladder cancer. In the patients of gallbladder cancer, the detection of a palpable mass in the right upper quadrant is not unique to cancer but it may be a significant sign since it may suggest the presence of a malignity [4, 8]. However, our patient had no palpable mass in the right upper quadrant.

The laboratory findings of the patients with no suspicion of cancer present no findings other than the findings of acute cholecystitis [7–9]. Similarly, our patient had no clinical or laboratory finding suggestive of cancer.

A number of studies have shown that terminating the surgical procedure may be beneficial in the patients who are preoperatively suspected with gallbladder cancer and undergo biopsy [6]. However, we preferred to continue the surgery since we had no available conditions for frozen biopsy during the surgery and the procedure was completed with no complication.

The staging of gallbladder cancer is performed according to the Japanese Biliary Surgical Society system [8]. Our patient presented with (R0) T1 N0 M0 squamous cell carcinoma with clean resection borders.

Previous studies have shown that gallbladder cancer should be suspected in the advanced-age patients admitted to hospital with the symptoms of cholecystitis. However, the localization and diameter of the tumor are a key factor for the prognosis of the cancer [2, 8]. The patients presenting with gallstones in the Hartman's pouch and proximal to the cystic duct present symptoms at early stages and thus enable early diagnosis and effective treatment [7, 8].

Squamous cell carcinoma of the gallbladder shows poor diagnosis, but since its overlap with cholecystitis presents early symptoms and thus leads to early diagnosis and effective treatment, the localization of the carcinoma is of prime importance [2, 8].

## 5. Conclusion

Gallbladder cancer should be kept in mind in the advanced-age patients presenting with the symptoms of cholecystitis.

## Figures and Tables

**Figure 2:**